# Comparison of the Cariogenic Potential of Two Sweeteners, Stevia and Aspartame, Based on Bacterial Inhibition and pH Reduction

**DOI:** 10.7759/cureus.100100

**Published:** 2025-12-25

**Authors:** Reshmi J, Shaniya Sain

**Affiliations:** 1 Department of Pediatric and Preventive Dentistry, Government Dental College, Thiruvananthapuram, Thiruvananthapuram, IND; 2 Department of Pediatric and Preventive Dentistry, PMS College of Dental Science and Research, Thiruvananthapuram, IND

**Keywords:** aspartame, hydrogen-ion concentration, stevia, sweetening agents, dental caries

## Abstract

Background and aim

Complete elimination of dietary sugars is impractical, especially for children. Restricting sugar intake and substituting sucrose with less cariogenic alternatives are plausible solutions. This in vitro study aimed to compare the cariogenic potential of two sweeteners, aspartame and stevia, by evaluating their bacterial inhibition capacity and the salivary pH changes produced at fixed time intervals.

Methods

To measure antibacterial activity, Petri plates containing 20 mL of Mueller-Hinton agar were seeded with a *Streptococcus mutans* culture adjusted to 0.5% McFarland Standard. Wells of approximately 10 mm diameter were bored using a well cutter, and different concentrations of the sweeteners (0.5%, 2.5%, 5.0%, and 10%) were added. Plates were incubated at 37°C for 24 hours, after which the zones of bacterial inhibition were measured. To measure pH changes, a test medium prepared by mixing artificial saliva (5 mL) with brain heart infusion medium (5 mL) was inoculated with *S. mutans* (50 µL). Samples were divided into two groups: stevia extract and aspartame. Increasing concentrations of both sweeteners were tested, and pH was recorded at regular intervals. Uninoculated test medium served as the control.

Results

Preliminary experiments were performed in single trials, followed by subsequent experiments carried out in triplicate to determine both pH changes and antibacterial activity of stevia and aspartame. The arithmetic mean of the observed values was used for statistical analysis. The zone of bacterial inhibition measured in millimeters and the pH change measured at varying concentrations of the two samples were compared using one-way ANOVA. The change in pH of various concentrations of stevia extract and aspartame recorded at different time intervals was compared using a paired t-test. The mean zone of bacterial inhibition was greater for stevia, and the area of inhibition increased with increasing concentration in both groups. No significant difference in pH change with increasing concentrations or time was observed between the aspartame and stevia groups.

Conclusions

Significant antimicrobial activity was exhibited by both aspartame and stevia against the cariogenic bacteria, *S. mutans*. The pH drop produced by both sweeteners over time also did not show significant differences under in vitro conditions. Hence, the findings suggest that aspartame and stevia possess comparable anticariogenic properties. Considering the calorific value and potential adverse effects of aspartame on human health, the natural sweetener stevia may be regarded as a better alternative.

## Introduction

Dental caries, the most common infectious disease affecting humans, is initiated by the localized action of acid produced when acidogenic microorganisms in the dental biofilm metabolize fermentable carbohydrates [1]. Mutans streptococci, particularly *Streptococcus mutans*, are frequently isolated from carious lesions and are considered the key bacteria involved in the etiology and progression of dental caries.

Free sugars are an essential dietary factor in the development of dental caries, as the disease does not occur in their absence. However, complete elimination of dietary sugars is impractical, especially among children. Restricting sugar intake and substituting sucrose with less cariogenic alternatives may help control caries initiation and progression. The inherent antimicrobial activity of such sugar substitutes would provide an added advantage in caries prevention. Consequently, ongoing research focuses on exploring novel approaches to combat cariogenic bacteria using natural products, including medicinal plants [2,3].

Aspartame (E951) is a synthetic dipeptide sweetener approximately 180-200 times sweeter than sucrose and has a low caloric value. It is widely used in over 6,000 commercial products worldwide under various brand names. Unlike sucrose, the microflora present in dental plaque cannot metabolize aspartame, which may be advantageous in preventing dental caries [4,5]. However, aspartame has been classified as possibly carcinogenic to humans (Group 2B) based on limited evidence for cancer in humans, specifically hepatocellular carcinoma. Although the Joint Expert Committee on Food Additives concluded that this evidence is not convincing, they recommend an acceptable daily intake of 0-40 mg/kg body weight [6].

Given these concerns, natural alternatives with no reported side effects are ideal, combining anticariogenic properties with safety. *Stevia rebaudiana *Bertoni is a plant that has gained attention in recent years due to its medicinal properties. It is a subtropical perennial shrub from the sunflower family (Asteraceae), native to Paraguay and Brazil, and is commonly called candyleaf, sweetleaf, or sugarleaf. Extracts obtained from the dried leaves contain glycosides approximately 200-300 times sweeter than sugar [7]. Stevia is a natural, calorie-free sweetener with good stability. It is reported to have various health benefits, including antidiabetic, antihypertensive, antioxidant, anti-inflammatory, antibacterial, and antiviral effects. It may also help control hyperlipidemia, obesity, and cancer and improve liver and kidney function. Importantly, no adverse effects on human health have been reported.

The cariogenic potential of sugars can be broadly assessed by examining their influence on oral bacteria and their impact on salivary pH. Foods that enhance the growth or acidogenic activity of cariogenic bacteria, or that produce a rapid or sustained drop in salivary pH, are more likely to promote demineralization and dental caries. Evaluating both bacterial inhibition capacity and salivary pH response provides a comprehensive understanding of how different foods contribute to oral health risk. This study was designed to compare, in vitro, the cariogenic potential of aspartame and stevia by evaluating their bacterial inhibition capacity and their effect on salivary pH over fixed time intervals.

## Materials and methods

Preparation of aspartame and stevia samples

Commercially available aspartame was procured from the local market, and the weight/volume concentrations used in the study were prepared in distilled water. To prepare stevia extract, dried leaves of *S. rebaudiana *(Asteraceae) were finely powdered. Fifty grams of stevia leaf powder were suspended in 100 mL of distilled water and maintained at 37°C for 24 hours. A 12% yield was obtained. The suspension was filtered through muslin cloth, and the extract was allowed to dry. A 0.01% solution of dimethyl sulfoxide was used as the vehicle control for the extract. Various concentrations of the dried stevia extract and aspartame were then prepared in distilled water for use in the study.

Measurement of pH change

Brain heart infusion (BHI) broth was used as the culture medium. It was prepared by dissolving 37 g of BHI broth (HiMedia Laboratories Private Limited, Mumbai, India) in 1,000 mL of distilled water and autoclaving at 121°C under 15 lb pressure for 15 minutes.

Artificial saliva was prepared by first dissolving 10 g of sodium carboxymethyl cellulose in 200 mL of boiling distilled water. The other components listed in Table 1 were dissolved in 600 mL of distilled water. Both solutions were then combined, and the pH of the mixture was adjusted to 6.75 using KOH. The final volume was made up to 1,000 mL with distilled water. The artificial saliva was autoclaved at 121°C under 15 lb pressure for 15 minutes and stored at 4°C until use.

**Table 1:** Composition of artificial saliva used in the study

No.	Composition	Weight/volume
1	Sodium carboxymethyl cellulose	10 g/L
2	KCl	0.625 g/L
3	MgCl₂·6H₂O	0.059 g/L
4	CaCl₂·2H₂O	0.166 g/L
5	K₂HPO₄	0.804 g/L
6	KH₂PO₄	0.326 g/L

To measure pH changes, the test medium was prepared by mixing 5 mL of artificial saliva with 5 mL of BHI medium and inoculated with the test bacteria (*S. mutans*, MTCC 890; growth adjusted to 0.5 McFarland Standard, equivalent to 1.5 × 10⁸ CFU/mL). All procedures were carried out under a laminar airflow hood.

**Figure 1:**
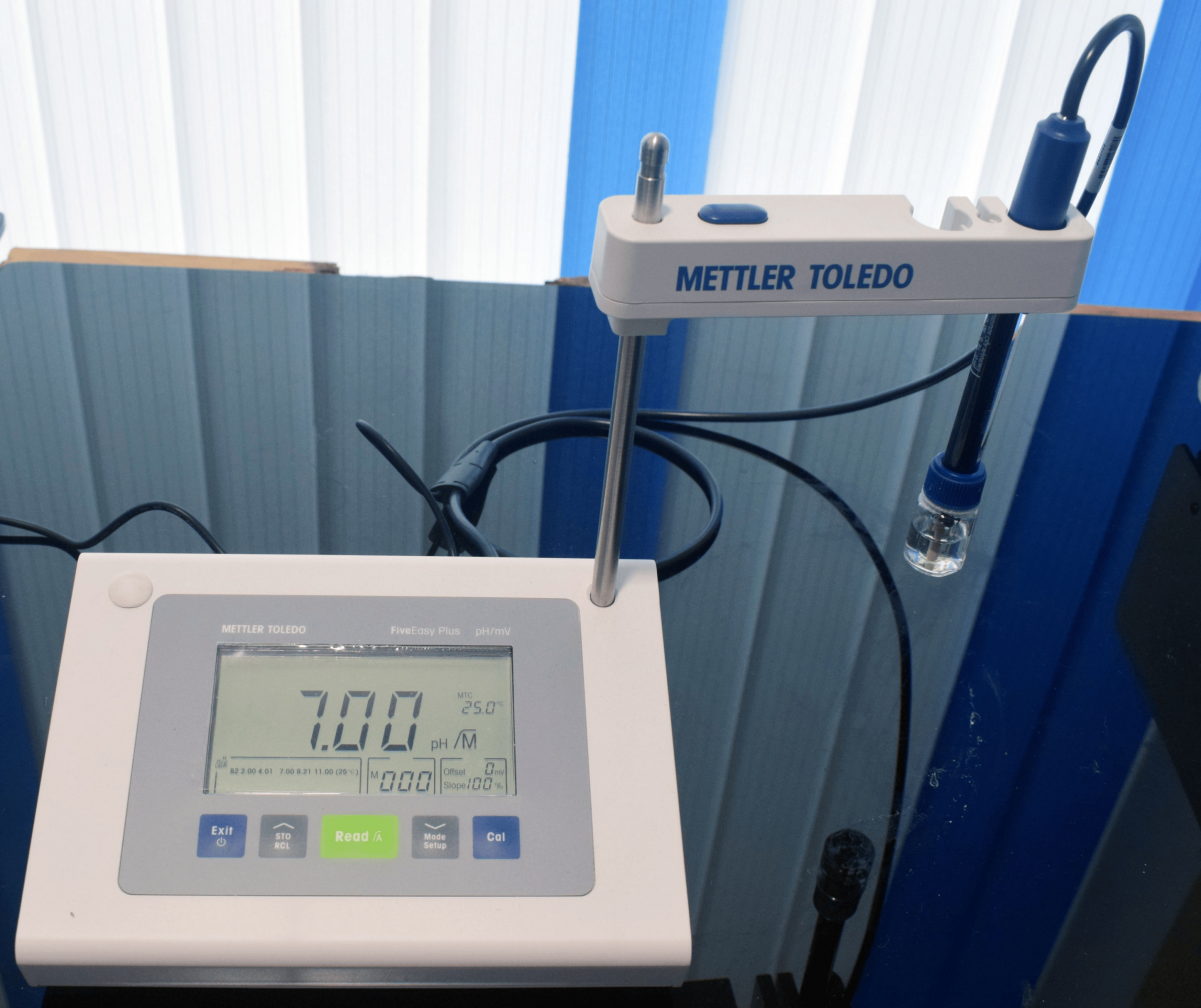
Digital pH meter

The test medium was divided into two sets: one for stevia extract and the other for aspartame. Stevia extract and aspartame were added at increasing concentrations of 0.5%, 2.5%, 5.0%, and 10%. Uninoculated test medium served as the control. The pH of the samples was measured using a five-point calibrated digital pH meter (Mettler Toledo, Columbus, OH, USA), calibrated with standard buffer solutions (pH 4.0, 7.0, and 10.0) prior to each experiment, at five minutes, 20 minutes, and one hour after inoculation (Figure 1).

Measurement of antibacterial activity

The antimicrobial activity was evaluated based on the principle that when antimicrobial agents in the samples diffuse into the culture medium of plates freshly seeded with test organisms, circular zones of inhibition are formed. The diameters of these zones were measured in millimeters.

Procedure

A pure culture of *S. mutans* (MTCC 890; growth adjusted to 0.5 McFarland Standard, equivalent to 1.5 × 10⁸ CFU/mL) was grown in nutrient broth prepared by dissolving 13 g of commercially available nutrient medium (HiMedia Laboratories Private Limited) in 1,000 mL of distilled water and boiling until fully dissolved. The medium was sterilized by autoclaving at 121°C under 15 lb pressure for 15 minutes. Petri plates containing 20 mL of this medium were seeded with the *S. mutans *culture.

**Figure 2:**
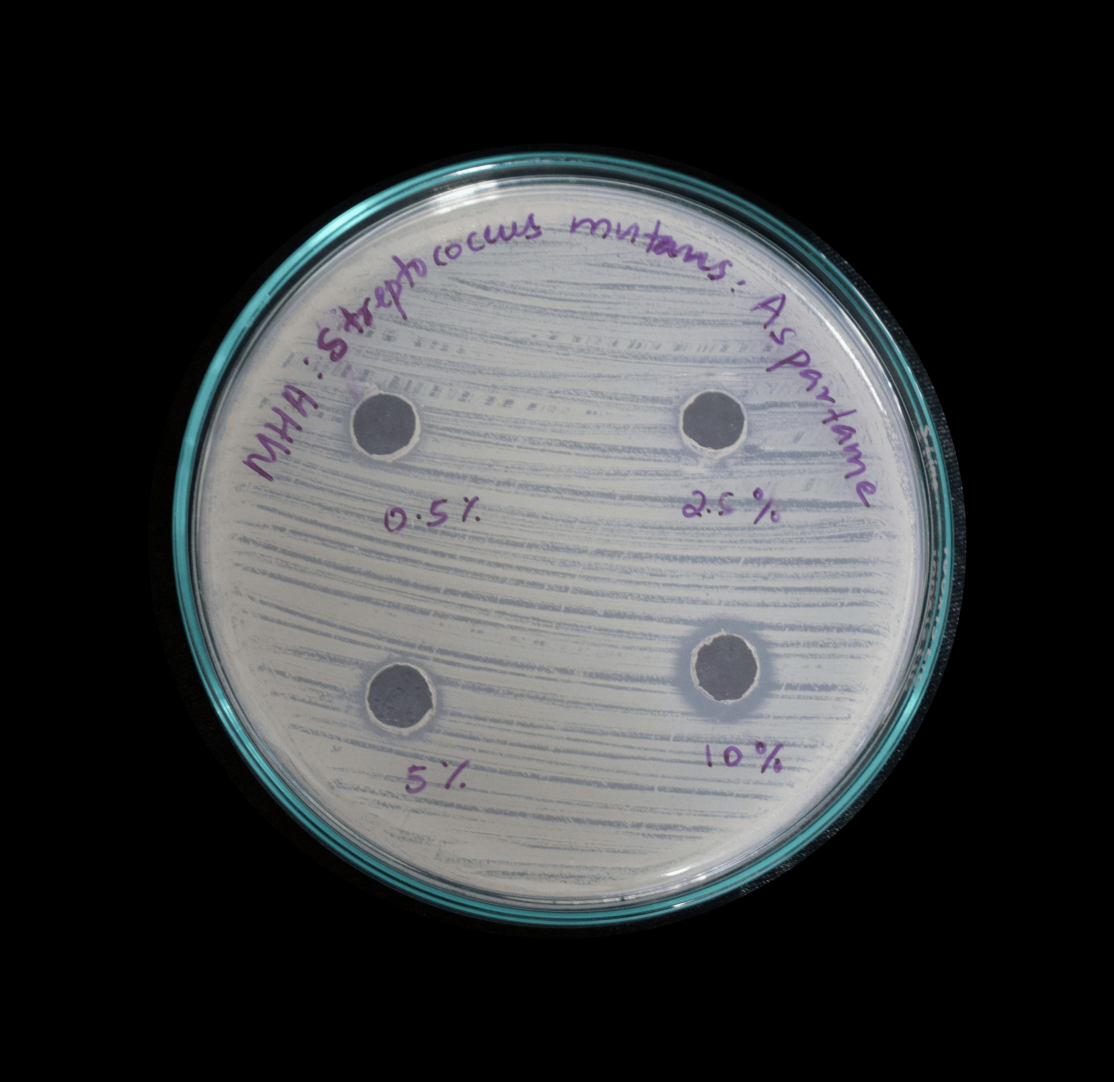
Zone of inhibition of aspartame

**Figure 3:**
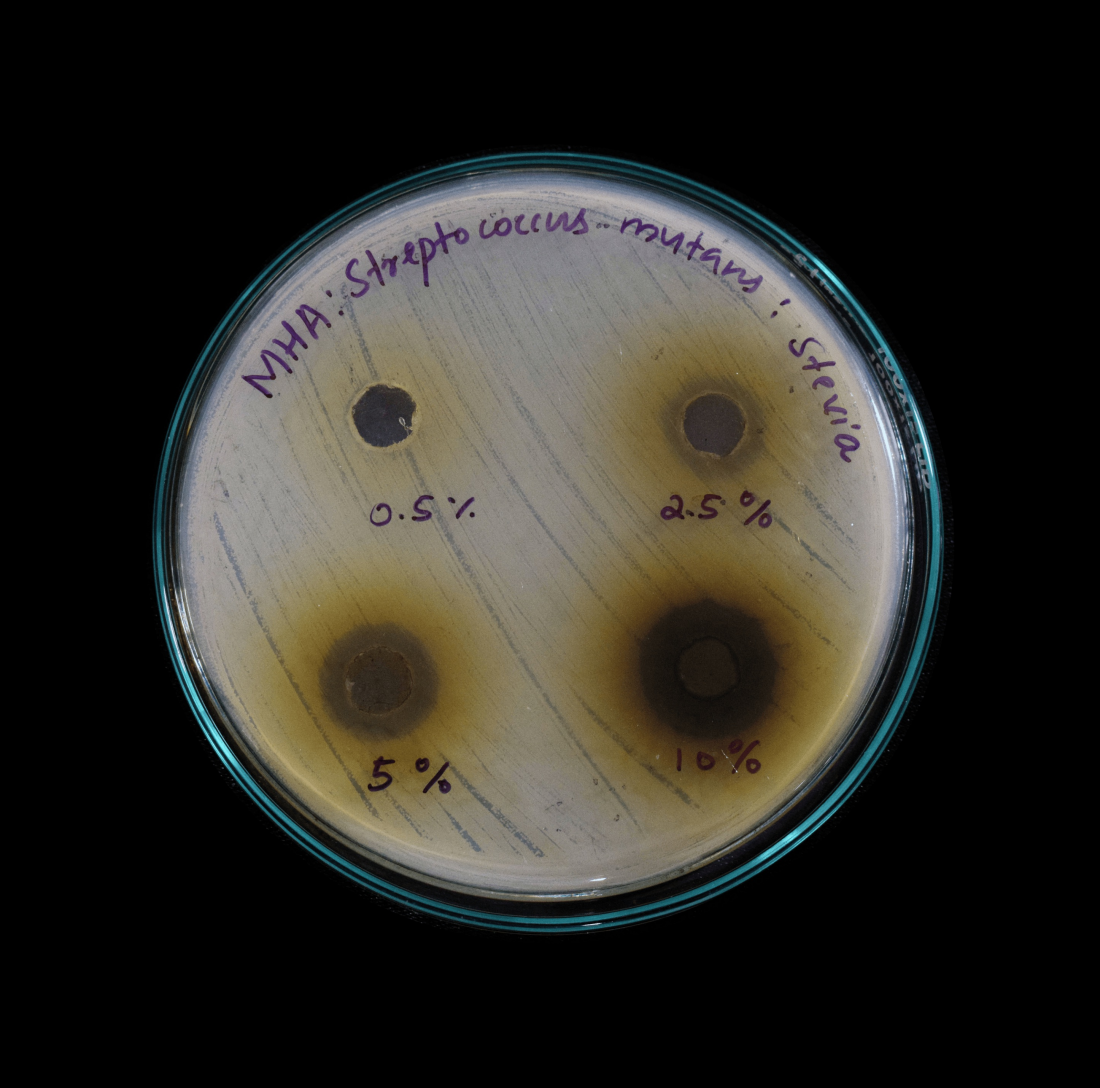
Zone of inhibition of Stevia rebaudiana

Mueller-Hinton agar medium was prepared by dissolving 33.8 g of the commercially available medium (MHI Agar) in 1,000 mL of distilled water. The medium was autoclaved at 121°C under 15 lb pressure for 15 minutes and poured while still molten into 100-mm Petri plates (25-30 mL/plate). Wells of approximately 10 mm were bored using a well cutter. Different concentrations of stevia and aspartame (0.5% (5 µg/µL), 2.5% (25 µg/µL), 5.0% (50 µg/µL), and 10% (100 µg/µL)) were added to the wells. Plates were incubated at 37°C for 24 hours, and antibacterial activity was determined by measuring the diameter of inhibition zones formed around the wells (NCCLS, 1993) (Figure 2, Figure 3).

**Figure 4:**
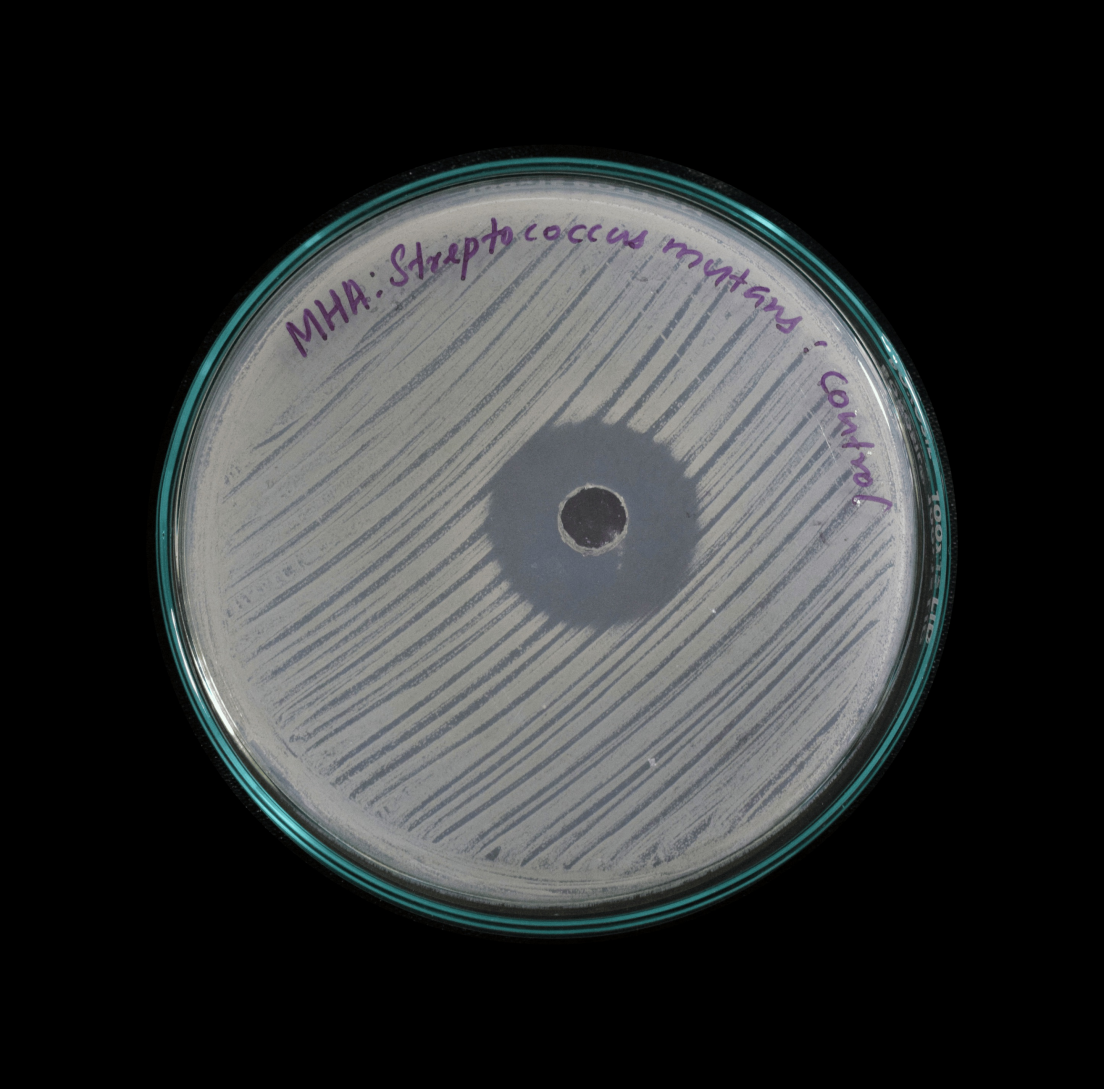
Positive control: streptomycin

Streptomycin was used as a positive control (Figure 4).

Preliminary experiments were performed in single trials, followed by subsequent experiments carried out in triplicate. The zone of bacterial inhibition (measured in millimeters) and pH changes at varying concentrations and time intervals for the two groups (aspartame and stevia) were compared using one-way ANOVA. Changes in pH for various concentrations of stevia extract and aspartame at different time intervals were compared using a paired t-test.

## Results

Experiments were performed in triplicate, repeating the procedures for three sets of samples of aspartame and stevia to determine both pH changes and antibacterial activity. The arithmetic mean of the observed values was used for statistical analysis. The pH changes produced by various concentrations of stevia extract and aspartame, compared with the control (uninoculated test medium), at five minutes, 20 minutes, and one hour are shown in Table 2 and Table 3, respectively.

**Table 2:** pH of increasing concentrations of stevia extract at various time intervals

Concentration of stevia extract (%)	Mean pH value after five minutes	Mean pH value after 20 minutes	Mean pH value after one hour
0.50%	7.4	7.4	7.4
2.50%	7.3	7.4	7.3
5.00%	7	7	7
10%	7	7	7

**Table 3:** pH of increasing concentrations of aspartame at various time intervals

Concentration of aspartame (%)	Mean pH value after five minutes	Mean pH value after 20 minutes	Mean pH value after one hour
0.50%	7.5	7.4	7.5
2.50%	7.4	7.4	7.4
5.00%	7	7	7
10%	7	6.9	6.9

Paired t-test analysis showed no significant change in pH values over time for either group (Table 4).

**Table 4:** Change in pH of various concentrations of stevia extract and aspartame at different time intervals

Group			Paired differences					t	df	Significance (two-tailed)
			Mean	SD	SEM	95% CI of the difference				
						Lower	Upper			
Stevia	Pair 1	Ph1-Ph2	-0.025	0.05	0.025	-0.10456	0.05456	-1	3	0.391
	Pair 3	Ph2-Ph3	0.025	0.05	0.025	-0.05456	0.10456	1	3	0.391
Aspartame	Pair 1	Ph1-Ph2	0.025	0.05	0.025	-0.05456	0.10456	1	3	0.391
	Pair 3	Ph2-Ph3	-0.025	0.05	0.025	-0.10456	0.05456	-1	3	0.391
	Pair 2	Ph1-Ph3	0	0.08165	0.04082	-0.12992	0.12992	0	3	1

The mean values of the zones of inhibition produced by various concentrations of stevia extract and aspartame are shown in Table 5 and Table 6, respectively.

**Table 5:** Measurement of the zone of bacterial inhibition of various concentrations of stevia extract

Sample	Concentration (µg)	Zone of inhibition (mm), Plate 1	Zone of inhibition (mm), Plate 2	Zone of inhibition (mm), Plate 3	Mean (mm)
Stevia	Streptomycin (100 µg)	28	28	28	28
	0.50%	Nil	Nil	Nil	0
	2.50%	10	12	14	12
	5%	12	11	16	13
	10%	20	20	20	18

**Table 6:** Measurement of the zone of bacterial inhibition of various concentrations of aspartame

Sample	Concentration (µg)	Zone of inhibition (mm), Plate 1	Zone of inhibition (mm), Plate 2	Zone of inhibition (mm), Plate 3	Mean (mm)
Aspartame	Streptomycin (100 µg)	28	28	28	28
	0.50%	Nil	Nil	Nil	0
	2.50%	10	12	11	11
	5%	11	12	13	12
	10%	14	12	13	13

No significant differences were observed between the two groups with regard to pH change over time or the zone of inhibition (Table 7, Table 8). The very low F-statistics indicate that the variance between groups (stevia vs. aspartame) is much smaller than the variance within each group for both pH change and measured zone of bacterial inhibition. Furthermore, p-values of 0.926 and 0.832 indicate that the differences in pH over time, as well as the zones of inhibition produced by the two sample groups, are not statistically significant.

**Table 7:** Comparison of the pH change produced by stevia extract and aspartame

pH	Sum of squares	df	Mean square	F	Significance
Between groups	0	1	0	0.009	0.926
Within groups	1.046	22	0.048		
Total	1.046	23			

**Table 8:** Comparison of the zone of inhibition of stevia extract and aspartame

Zone of inhibition	Sum of squares	df	Mean square	F	Significance
Between groups	4.9	1	4.9	0.048	0.832
Within groups	811.6	8	101.45		
Total	816.5	9			

## Discussion

In this study, we compared the cariogenic potential of a commonly used artificial sweetener, aspartame, with its natural alternative, *S. rebaudiana *Bertoni. Both sweeteners exhibited significant and comparable antibacterial activity, suggesting that they can help reduce *S. mutans *counts in the oral cavity. The pH of the test medium remained near the physiological pH of saliva in both samples during the first hour, indicating that both aspartame and stevia have significant anticariogenic properties. However, the small sample size of this study was insufficient to detect any subtle differences between the two sweeteners.

Since the role of sucrose and related sugars in caries activity was established a century ago, researchers have sought non-acidogenic sugar substitutes that do not reduce the pH of dental plaque. By the late 20th century, artificial sweeteners such as aspartame began to be used as substitutes for sucrose, the “archcriminal” of dental caries [8]. Their intense sweetness allows smaller quantities to achieve the desired level of sweetness, increasing demand in body weight management, diabetes control, the food and beverage industry, oral health products, and various pharmaceuticals [9]. Consumption of low-calorie sweeteners has increased markedly over the past several decades in developed countries, including the United States [10]. Limited data on artificial sweetener use in rapidly developing countries, such as India, do not rule out their widespread consumption.

While these artificial sweeteners are useful in caries prevention, their potential limitations from nutritional, toxicological, and economic perspectives are often overlooked. Reported adverse effects include gastrointestinal disturbances due to altered gut microflora, changes in neurologic responses and taste perception, allergic reactions, insulin and metabolic derangements, and cardiovascular effects. Several studies have explored potential associations between artificial sweetener use and cancer development [11,12]. Although the earliest studies suggested a positive association between artificial sweeteners and bladder cancer risk in males, subsequent studies failed to confirm this finding [13-15]. However, recent evidence indicates a potential association between higher consumption of artificially sweetened beverages and renal cancer risk in postmenopausal women [16]. Formaldehyde, a metabolic byproduct of aspartame, is a known carcinogen that can cause DNA damage, chromosomal aberrations, and mitotic errors, raising concerns about aspartame’s long-term safety [17]. Some animal studies have also demonstrated carcinogenic effects of aspartame at multiple sites in rodents, reinforcing concerns about its potential risk in humans [18].

Considering these potential health hazards, naturally available alternatives with equivalent or better sweetening properties, anticariogenic effects, and minimal toxicity are preferable. *S. rebaudiana *Bertoni is 200-300 times sweeter than sucrose, with no known adverse effects on human health [19]. It also confers beneficial systemic effects and protective effects against caries and periodontal diseases [20]. Stevia extracts prepared in different solvents have demonstrated significant antimicrobial activity against various fungi and both gram-positive and gram-negative bacteria, including *S. mutans *and *Lactobacillus acidophilus*. The aqueous extract of stevia leaves shows potent antibacterial activity, while the methanolic extract exhibits strong antifungal activity [21]. Its activity against food-spoiling fungi such as *Aspergillus niger* and bacteria such as *Staphylococcus aureus* supports its potential use as a preservative in the food industry [22].

Beyond its antimicrobial properties, stevia leaf extract exhibits antioxidant, anti-inflammatory, anti-obesity, and anticancer effects [23]. *S. rebaudiana *Bertoni may also serve as part of a plant-based diet for cancer prevention or adjunct therapy alongside conventional treatments [24]. These health benefits are attributed to bioactive compounds in stevia leaves, including phenols, flavonoids, sterols, terpenes, tannins, vitamins, and minerals [25].

In this study, both sweeteners were evaluated for antibacterial activity against *S. mutans* and their influence on the pH of the test medium. While both aspartame and stevia demonstrated zones of inhibition indicative of antibacterial activity, no statistically significant differences were observed between the groups. Similarly, pH levels remained within the physiological range of saliva during the first hour, with no significant alterations noted between treatments.

These findings suggest that both sweeteners may exhibit anticariogenic potential, particularly in reducing the viability of cariogenic bacteria under controlled in vitro conditions in agar diffusion assays. However, the limited sample size likely constrained the ability to detect subtle effects and account for natural biological variability. Comprehensive investigations are needed to determine the minimum inhibitory concentrations of both agents and to elucidate their effects on oral biofilms, which are the key ecological niches for cariogenic bacteria. Furthermore, the ex vivo design does not replicate the complexity of the oral environment, where factors such as salivary flow and composition, host immune responses, microbial interactions, and metabolic and hormonal influences critically modulate cariogenicity.

Future research should involve larger, well-powered in vivo studies in human subjects to validate these preliminary observations and clarify the mechanisms underlying the anticariogenic effects of these sweeteners. Such studies will be essential to establish clinically relevant conclusions and inform evidence-based recommendations for their use in oral health management.

Given the equivalent anticariogenic potential of the two sweeteners, their impact on overall human health becomes the major criterion when choosing between stevia and aspartame as alternatives to sucrose. The superior safety profile and beneficial systemic effects of stevia compared with aspartame have been demonstrated in previous studies. The formation of toxic metabolites after aspartame ingestion raises concerns regarding its long-term safety. The anticariogenic effects of stevia in humans have not been sufficiently investigated; hence, further human trials are warranted to determine its role in promoting oral health through potential antibacterial effects.

## Conclusions

The search for an anticariogenic and safe alternative to sucrose has long been the cornerstone of research in cariology. The limited acceptance of many artificial sweeteners is largely attributed to their potential adverse effects, including concerns regarding carcinogenicity. In the present study, both aspartame and stevia demonstrated significant antimicrobial activity against the cariogenic bacterium S. mutans under in vitro conditions. No statistically significant differences in pH reduction were observed between the two sweeteners when inoculated into S. mutans cultures. These findings suggest that aspartame and stevia possess comparable anticariogenic properties. However, when considering caloric content and the potential adverse health effects associated with aspartame, the natural sweetener stevia may be regarded as a safer and more favorable alternative, particularly for use in children.
